# IN HIP FRACTURE, GENDER CONFOUNDS COGNITION ASSESSMENT, TIME TO DEATH, AND COGNITION-RELATED MORTALITY

**DOI:** 10.1093/geroni/igz038.1788

**Published:** 2019-11-08

**Authors:** Heather L Mutchie, Denise Orwig, Ann Gruber-Baldini

**Affiliations:** University of Maryland Baltimore School of Medicine, Baltimore, Maryland, United States

## Abstract

Of 300,000 annual hip fractures in the US, about 30% occur in men, over 30% experience cognitive impairment or dementia, and 30% die within one year. This study compares time-to-death and cognition-related cause of death (CR-COD) by gender after hip fracture using different methods of cognitive impairment ascertainment. Baseline hospital charts and Modified Mini-Mental State Examination (3MS) were from the Baltimore Hip Studies 7th cohort (2006-2011) (171 women, 168 men). National Death Index was obtained up to December 31, 2014. Cox models assessed dementia diagnosis in chart and 3MS<78 as predictors of mortality and CR-COD (died n=204). Men were more likely to fail the 3MS (Men:26.2%, Women:16.2%; p=0.025), die (Men:73.8%, Women:46.8%; p<.0001), and die sooner (Men:µ=30.8months, Women:µ=39.2months; p=0.0159). Significantly more women died with CR-COD (Men:17.7%, Women:31.2%, p=0.0253). Among men, those with any cognitive impairment (3MS:µ=21.5months, chart:µ=21.3months; both:µ=22.0months) died sooner than non-impaired (µ=22.0months). Among women only those with both chart and 3MS (µ=16.7months) died sooner than all other groups (3MS:µ=47.8months, chart:µ=35.1months, none:µ=42.9months). With respect to CR-COD, men with cognitive impairment from any source (3MS:µ=5.0months, chart:µ=43.4months, both:µ=30.5months) differed from non-impaired (µ=91.6months). While women with hospital chart alone (µ=14.0months) differed from non-impaired (µ=3.6months), 3MS alone (µ=8.5months) and both (µ=9.5months). Men had worse 3MS scores but similar chart diagnosis, indicating a potential underdiagnosis of men’s cognitive impairment. Gender confounds the relationship between cognition and death or CR-COD. In men, any impairment predicts death overall and CR-COD. In women, chart with 3MS impairment predicted overall death, but chart alone was more predictive for CR-COD.

